# Lenvatinib Plus Pembrolizumab Versus Chemotherapy in Advanced Endometrial Cancer: Efficacy and Safety Insights

**DOI:** 10.7759/cureus.89030

**Published:** 2025-07-30

**Authors:** Faizah Alabi, Izuchukwu F Okpalanwaka, Bryant Azoroh, Ebenezar Okoyeocha, Temilola Balogun, Idris B Ayinde

**Affiliations:** 1 Department of Immunotherapeutics and Biotechnology, Texas Tech University Health Sciences Center, Abilene, USA; 2 Oncology Research Group, Tropical Medical Oncology Awareness Foundation, Lagos, NGA; 3 Department of Immunotherapeutics and biotechnology, Texas Tech University Health Sciences Center, Abilene, USA; 4 Pharmacology and Toxicology, Michigan State University, Michigan, USA; 5 Center for Parasitology and Disease Research, School of Environmental Sciences, University of Salford, Salford, USA; 6 Pharmaceutical Chemistry, University of Lagos College of Medicine, Lagos, NGA; 7 Pharmacy, Lifeline Pharma Limited, Lagos, NGA

**Keywords:** advanced endometrial cancer, chemotherapy, immune checkpoint inhibitors, lenvatinib, mismatch repair deficient, mismatch-repair proficient, pembrolizumab, targeted therapy

## Abstract

Advanced endometrial cancer (aEC) presents a formidable therapeutic challenge, particularly in patients with recurrent or metastatic disease. Historically, platinum-based chemotherapy is the mainstay treatment for aEC. However, the treatment paradigm has shifted with the emergence of immune checkpoint inhibitors (ICIs) and targeted therapies. Lenvatinib combined with pembrolizumab (LVB + PMB) has emerged as a promising regimen, particularly for patients with proficient mismatch repair (pMMR) tumors who typically respond poorly to ICI monotherapy. This review synthesizes recent data comparing LVB + PMB to chemotherapy, focusing on efficacy, safety, and molecular subtype-guided treatment selection. Findings from the KEYNOTE-775 and LEAP-001 trials are highlighted. We also delve into the molecular and immunologic landscape of aEC, providing a mechanistic rationale for treatment response and resistance. While LVB + PMB shows superior progression-free survival in second-line settings, its first-line application in unselected populations remains inconclusive. Therefore, strategic patient stratification and biomarker development remain key to maximizing clinical outcomes. Furthermore, we discuss the clinical implications of these findings, explore future research directions, including novel combinations and biomarkers, and provide recommendations for the evolving therapeutic landscape.

## Introduction and background

Endometrial cancer (EC), also known as uterine cancer, is the most frequently occurring gynecological cancer in advanced nations like the United States, and its worldwide prevalence is particularly high in obese and younger populations [1,2]. In the United States, 66,200 new cases of EC were estimated in 2023, with ~13,000 deaths [3]. While early stage disease is often curable with surgery, adjuvant radiotherapy, or chemotherapy to reduce recurrence after surgery, advanced and recurrent disease pose a significant treatment challenge [4,5]. The five-year survival rate is currently below 20% for advanced (categorized as stage III and IV according to the International Federation of Gynecology and Obstetrics) or metastatic endometrial cancer, underscoring the need for effective systemic therapies. Historically, platinum- and taxane-based chemotherapy have been the first-line treatment for advanced, metastatic, or recurring EC. However, their effectiveness and safety remain mainly underwhelming [6]. While chemotherapy can induce a modest response in patients, its benefits are often limited by short response durations and significant toxicity [7].

Recent advances in molecular classification and immunologic profiling have revolutionized our understanding of EC biology and spurred the development of targeted and immune-based therapies. Among these are immune checkpoint inhibitors (ICIs), which have greatly improved patients' outcomes in the clinical oncology of many solid tumors. The Cancer Genome Atlas (TCGA) project has classified four molecular subtypes of EC (POLE-ultramutated, microsatellite instability-high (MSI-H)/mismatch repair-deficient (dMMR), copy-number low, and copy-number high). Tumors with dMMR/MSI-H subtypes generally respond to ICIs [8,9]. Notably, around 20-40% of all ECs are dMMR, representing the highest prevalence of dMMR among solid tumors [10,11]. By contrast, mismatch-repair proficient (pMMR) tumors (often copy-number high TP53-mutant or no specific molecular profile [NSMP]) are immune-cold and respond poorly to ICI. This dichotomy underscores a new risk-adapted therapeutic approach, leading to ICI (pembrolizumab [PMB] or dostarlimab) as a monotherapy for dMMR, and novel combinations were developed for pMMR aEC.

The combination of lenvatinib (LVB), a tyrosine kinase inhibitor (TKI) that targets multiple kinases such as vascular-endothelial growth factors 1-3 (VEGF1-3), fibroblast growth factor receptor 1-4 (FGFR 1-4), platelet-derived growth factor receptor ⍺ (PDGFR⍺), KIT, and RET, with both antiangiogenic and immunomodulatory effects [12], and PMB, a monoclonal antibody that blocks programmed death-1 (PD-1) to reactivate the anti-tumor T cell responses, has emerged as a promising regimen, with studies indicating clinical benefits in pretreated aEC patients, particularly pMMR tumors for which single-agent ICIs yielded modest responses. This led to the Food and Drug Administration (FDA) approval as a second-line treatment for advanced, previously treated pMMR aEC patients [12,13]. Given the rapid evolution of this field, we present a comprehensive review of LVB + PMB versus standard chemotherapy in aEC, regarding efficacy, safety, molecular and mechanistic context, and implications for clinical practice and research. We also highlight key clinical trials (e.g., NRG-GY018, RUBY, KEYNOTE-775, ENGOT-EN9/LEAP-001), emerging biomarkers, and combination strategies and suggest avenues for future investigations and insights.

## Review

Molecular and immunologic landscape of endometrial cancer

EC is a heterogeneous malignancy characterized by diverse molecular and immunologic profiles, which have critical implications for patient prognosis, treatment strategies, and clinical outcomes. A significant challenge in the management of advanced endometrial cancer (aEC) is the inadequacies in effectively diagnosing and predicting the responsiveness of patients to various treatment modalities [14]. Previous and most common diagnostic approaches for aEC primarily rely on histopathological and histomorphological features, which are often unreliable and poorly reproducible due to tumor grade heterogeneity [15]. These inconsistencies in diagnosis can lead to inappropriate patient stratification, resulting in suboptimal treatment regimens that adversely affect therapeutic efficacy and patient survival. To address these challenges, The TCGA project has identified and characterized four molecular subtypes of EC based on genetic and molecular features, utilizing advanced next-generation sequencing techniques [16]. This initiative encompasses comprehensive genomic, proteomic, and transcriptomic analyses of 373 EC patients. The Proactive Molecular Risk Classifier for Endometrial Cancer (ProMisE) and the Translational Research in Post-operative Radiation Therapy in EC (TransPORTEC) classifiers have validated these findings [17]. Integrating these parameters into clinical practice is poised to enhance the understanding of tumor behavior and response to therapy, particularly in immunotherapy and targeted therapy.

The POLE ultramutated (POLEmut) subtype is characterized by extensive mutations in the *POLE* gene, a component of the catalytic subunit of deoxyribonucleic acid (DNA) polymerase epsilon. This enzyme is essential for DNA replication and repair, thereby playing a critical role in maintaining DNA fidelity [16,18]. Given its vital function, a mutation in this gene is regarded as a driver mutation. Such mutations are characterized by high neoantigen load and increased immune infiltration, making them a promising biomarker for immunotherapy responsiveness, which is associated with increased immunogenicity and a more favorable prognosis for treatments such as ICIs [19]. Another related classification is MSI-H or dMMR, which similarly exhibits high immunogenicity and neoantigen load, resulting in enhanced responsiveness to immunotherapy [20]. These tumors are known to be deficient in the DNA mismatch repair system. They are also classified as immunologically hot due to high tumor-infiltrating lymphocyte (TIL) and elevated immune checkpoints such as PD-1/programmed death ligand-1 (PD-L1), contributing to their response to ICIs [21]. Clinically, dMMR aECs respond remarkably to ICIs, with an objective response rate of 48% progression-free survival (PFS) of 13.1-24 months. This led to regulatory approval of ICIs for dMMR/MSI-H aEC in both frontline (combined with chemotherapy) and recurrent settings [22,23]. However, PD-L1 as a biomarker of immunotherapy responsiveness is still being evaluated due to its high heterogeneity within the tumor [24].

Copy-number low (NSMP) endometrioid tumors are recognized as exhibiting low copy number alterations, thus resulting in a low tumor mutational burden (TMB), but they do not possess distinct molecular characteristics. These tumors are frequently characterized by gene mutations that drive endometroid cancer pathogenesis, including phosphatase and tensin homolog (PTEN), Kirsten rat sarcoma viral oncogene (KRAS), adenine thymine-rich interactive domain 1A (ARID1A), and phosphatidylinositol-4,5-bisphosphate 3-kinase catalytic subunit alpha (PIK3CA). Additionally, they are associated with a favorable prognosis, particularly in response to hormonal or targeted therapy [25]. In contrast, copy-number high tumors present elevated copy number alterations, contributing to high genomic instability and aberrant tumor protein 53 (TP53) expression. This subtype is associated with poor prognosis and reduced responsiveness to immunotherapy [26]. The MITO END-3 trial revealed that p53 mutations are associated with worse response to avelumab, a PD-L1 inhibitor [27]. However, they can benefit from targeted therapies such as poly(ADP-ribose) polymerase (PARP) inhibitors or anti-human epidermal growth factor receptor 2 (anti-HER2) agents [28]. NSMP and p53-mutant tumors are known to exhibit low TIL infiltration and a highly immunosuppressive tumor microenvironment (TME), often due to factors such as stromal cells, particularly vascular endothelial growth factor-mediated angiogenesis, defective dendritic cells (DCs), increased regulatory T cells (Tregs), and M2-macrophage polarization, which can impede immune cell infiltration [28].

Beyond these four subtypes, other mutations, including PTEN and ARIDIA, are being investigated and have been found to be associated with a positive response to immunotherapy. The MITO END-3 trial reported that patients with such mutations exhibited improved PFS when treated with avelumab, suggesting that these mutations may serve as biomarkers for selecting patients who could benefit from immunotherapy [27]. Mutations in breast cancer genes (BRCA1/2) are also observed in patients with aEC, albeit less frequently [29,30]. Their role in aEC is currently being examined in the context of PARP inhibitors and other targeted therapies. Hormone receptor status, such as estrogen and progesterone receptors, represents established prognostic markers in aEC [31]. Hormone-positive tumors may derive benefit from endocrine treatment, utilized in conjunction with chemotherapy or targeted therapy [32]. HER2 overexpression is frequently associated with aggressive disease in aEC patients. Such patients may benefit from HER2-targeted therapies, including trastuzumab [33].

**Figure 1:**
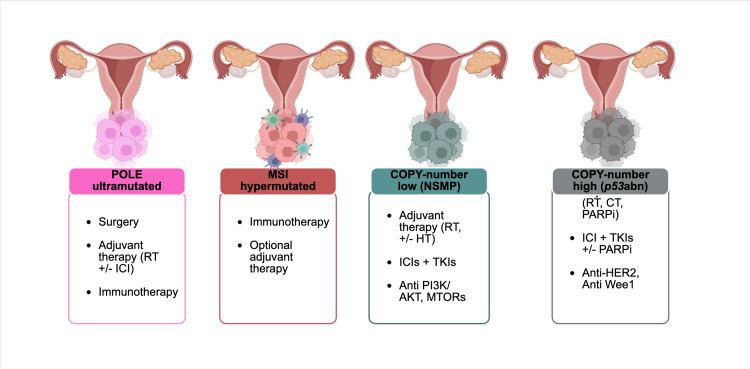
The molecular classification of endometrial cancer according to TCGA’s ProMisE TCGA identifies four distinct subtypes: POLE-ultramutated, MSI-hypermutated, copy-number low or NSMP, and copy-number low or p53-absent (p53abn). Each subtype plays a significant role in shaping treatment approaches, as summarized in the accompanying box, thereby enhancing efficacy and reducing toxicity. This is an original figure made by the authors using BioRender. TCGA, The Cancer Genome Atlas; ProMisE, Proactive Molecular Risk Classifier for Endometrial Cancer; MSI, microsatellite instability; NSMP, no specific molecular profile

Furthermore, DNA methylation has been investigated as a potential biomarker in aEC. Hypermethylation of genes such as mutL homolog 1 (MLH1) has been associated with microsatellite instability (MSI) and may serve as a complementary biomarker for immunotherapy [34,35]. Recently, studies have identified immune-related long non-coding RNAs (lncRNAs) that could predict responses to immunotherapy [36]. Consequently, a 13-lncRNA signature has been developed to classify patients into high-risk and low-risk groups, with low-risk patients demonstrating a better response to immunotherapy [37]. The dysregulation of signaling pathways such as mammalian target of rapamycin (mTOR), phosphoinositide 3-kinase/protein kinase B (PI3K/AKT), PIK3CA, and PTEN can benefit from targeted therapies, including mTOR inhibitors and PI3K/AKT inhibitors, as such mutations have been associated with sensitivity to AKT inhibitors. These findings underscore the emerging paradigm of personalized medicine in EC, integrating molecular diagnostics to guide therapy selection. The summary of the four subtypes is highlighted in Figure 1.

Chemotherapy in advanced endometrial cancer

Chemotherapy has historically constituted the standard of care as the first-line treatment for advanced or metastatic endometrial carcinoma. The standard regimen involves platinum-based regimens, notably the combination of carboplatin and paclitaxel every 21 days, which have been widely employed in therapeutic settings. This regimen has consistently demonstrated significant efficacy compared to alternative chemotherapeutic agents, with reported response rates (RRs) ranging from 40% to 60% across multiple studies [38]. Various chemotherapeutic agents have been explored as monotherapies and combination therapies. Specifically, methotrexate exhibited a modest RR of 6% [39], while docetaxel presented an RR of 31%[40]. Notably, esorubicin was associated with a lack of measurable response and notable toxicity, affecting 60% of treated patients [41]. Doxorubicin in combination with cyclophosphamide yielded a RR of 31% [42], and the duo of doxorubicin and cisplatin recorded a notable RR of 92% in treatment-naïve patients, contrasted by a 50% RR in patients with prior treatment histories; however, the duration of response (DoR) was comparatively brief at 10 months [38,42].

In contrast, the carboplatin-paclitaxel regimen not only provided a longer DoR but was also associated with an overall survival (OS) period extending from 12 to 29 months [43]. As a result, the National Comprehensive Cancer Network (NCCN) guidelines strongly recommend implementing a carboplatin and paclitaxel combination therapy over a 21-day cycle. Furthermore, this regimen may be effectively combined with trastuzumab in patients exhibiting human epidermal growth factor-2 (HER2) positivity [44]. Other treatment options, including combinations of bevacizumab with carboplatin and docetaxel or cisplatin in conjunction with docetaxel, may also be considered based on individual patient factors and clinical presentations.

The advent of immunotherapy has shifted the treatment paradigm for aEC. Incorporating ICIs such as PMB or dostarlimab to augment chemotherapy has markedly improved patient outcomes [45]. For example, the NRG-GY018 trial revealed that the addition of PMB to the carboplatin and paclitaxel regimen resulted in a 70% decrease in the risk of disease progression or mortality among patients with dMMR tumors [46]. Similarly, the RUBY trial delineated that incorporating dostarlimab in conjunction with standard chemotherapy yielded improvements in PFS for both dMMR and pMMR patients [47,48]. These breakthroughs have established chemo-immunotherapy combinations as a new second-line or other-line treatment for pMMR aEC with a potential superior benefit for dMMR patients.

Immunotherapy and targeted therapy in advanced endometrial cancer

Immunotherapy, particularly using ICIs, has emerged as a transformative approach in treating aEC. ICIs directed towards PD-1/PD-L1 axis have shown exceptional efficacy, especially in patients with dMMR tumors [49]. Initially, patients with advanced or recurrent EC are usually placed on systemic palliative chemotherapy as a front-line option and no standard-of-care second-line option [38]. This is due to the development of chemoresistance and heightened toxicity, surpassing these therapies' efficacious benefits.

Implementing immune ICIs for EC commenced with the approval of PMB for tumors exhibiting MSI-H or dMMR characteristics in recurrent settings in 2017, following the unprecedented responses documented in refractory dMMR cancers. Specifically in EC, PMB demonstrated an ORR and PFS of 48% and 13.1 months, respectively, coupled with a four-year DoR of 66% and an OS rate of 59% [50]. By contrast, RRs in pMMR cancers were reported to be less than 15%. Dostarlimab, another PD-1 inhibitor, likewise garnered FDA approval for dMMR recurrent EC, with an ORR of 45% in the GARNET trial [51]. These ICIs have dramatically improved outcomes for dMMR EC, which historically respond poorly to chemotherapy. Tumors with dMMR or MSI-H are characterized by a high mutational burden and increased PD-L1 expression, rendering them more susceptible to immunotherapy [50,52,53].

The efficacy of single-agent ICIs in treating pMMR aEC is notably limited. Consequently, alternative strategies have been explored, particularly combining ICIs with other therapeutic modalities to counteract the immunosuppression in the TME of pMMR tumors. One such approach involves the combination of LVB and PMB, demonstrating a synergistic effect by targeting the cancer through anti-angiogenesis and immune activation. The success of this combination therapy was observed in phase II studies, yielding an ORR of 38% in pMMR patients, significantly surpassing the ORR of individual agents within this population. This promising outcome prompted a phase III trial, ultimately leading to the approval of LVB + PMB as a second-line therapy for aEC, specifically for pMMR tumors following prior chemotherapy [54,55]. In the ensuing sections, we elaborate on the efficacy and safety of this combination compared to chemotherapy.

Ongoing evaluations are exploring various targeted therapies, including PARP inhibitors such as olaparib, to be used as maintenance treatment alongside durvalumab and chemotherapy as separate arms in the DUO-E trial [56]. Also, under consideration is anti-HER2 therapy, specifically trastuzumab, for patients with HER2-positive serous carcinoma [57]. New ICIs beyond the PD-1 pathway, such as PD-L1, cytotoxic T-lymphocyte antigen 4 (CTLA-4), and lymphocyte-activation gene 3 (LAG-3) inhibitors, are also being examined. For example, the PD-L1 inhibitor avelumab was assessed for its effectiveness in the maintenance settings. Although the overall outcomes were modest, molecular analysis from the MITO END-3 trial indicated that specific mutations, such as those in PTEN and ARID1A, could be associated with a more favorable response to avelumab.

In contrast, mutations in TP53 seemed to indicate resistance [27]. Moreover, trials involving combination immunotherapies are underway; for instance, an early phase study of a bispecific PD-1/CTLA-4 antibody, cadonilimab, in conjunction with LVB showed promising initial results in Chinese patients [58]. These initiatives demonstrate an expanding therapeutic landscape focused on customizing immune-targeted combinations based on tumor biology. A summary of ongoing and completed trials involving LVB and other targeted therapies in aEC is detailed in Table 1 [59-66].

**Table 1:** Key outcomes of chemotherapy, immunotherapy, and targeted therapy-containing regimens in advanced endometrial cancer CR, complete response; DCR, disease control rate; DoR, duration of response; dMRR, mismatch repair-deficient; EC, endometrial cancer; FDA, Food and Drug Administration; ICI, immune checkpoint inhibitor; LAG-3, lymphocyte activation gene 3; mOS, median overall response; mPFS, median progression-free survival; ORR, overall response rate; OS, overall response; PARP, poly(ADP-ribose) polymerase; PD-L1, program-death death ligand-1; PFS, progression-free survival; pMMR, mismatch repair-proficient; RR, response rate; TKI, tyrosine kinase inhibitor

Treatment Regimen	Key Efficacy Outcomes (Advanced ECs)	Clinical ID	Reference(s)
Carboplatin + paclitaxel (first line)	ORR ~50%; median OS ~15–29 months; standard of care first-line chemo	NCT00063999	[43]
Doxorubicin + cisplatin (historical)	ORR 92% in chemo-naïve (50% if pretreated); short median response ~10 months	Nil	[59]
Doxorubicin + cyclophosphamide	ORR ~31% in advanced disease	Nil	[60]
Docetaxel (single agent)	ORR ~30%, PR ~23% in advanced or metastatic EC	Nil	{40]
Methotrexate (single agent)	RR ~6% (minimal activity)	Nil	[39]
Carboplatin + paclitaxel + trastuzumab	Improves PFS and OS in HER2-positive uterine serous carcinoma (mPFS: 29.6 months, mOS: 12.9 months) vs chemo alone (mPFS: 8 months, mOS: 24.4 months)	NCT01367002	[57]
Chemo + PMB (ICI)	Significantly improves PFS and OS in dMMR tumors (NRG-GY018: ~70% risk reduction); modest benefit in pMMR (HR ~0.54). Now a new standard for dMMR aEC.	NCT03914612	[61]
Chemo + dostarlimab (ICI)	Improves PFS in both dMMR and pMMR (PFS: 61.4%); OS (71.3%); led to FDA approval (dMMR)	NCT03981796	[47]
PMB + LVB (ICI+TKI)	Approved 2L regimen for pMMR aEC; superior PFS and OS vs chemo in prior-treated patients (KEYNOTE-775); did not improve PFS in pMMR when given 1L vs chemo (LEAP-001)	NCT03517449, NCT04865289	[62–64]
LVB only	mPFS 5.4 months, mOS 10.6 months, ORR 61%	NCT01111461	[65]
Avelumab (PD-L1) + chemo (investigational)	Shown to improve PFS in a molecularly selected subset (dMMR); p53-mutant tumors had poorer response	NCT03503786	[27]
Durvalumab + chemo ± olaparib	Under investigation in first line (DUO-E trial). Initial results: adding durvalumab improves PFS; adding a PARP inhibitor did not significantly augment benefit	NCT0426920	[56]
LVB + paclitaxel	PFS: 65%, ORR: 65%	NCT02788708	[65]
PMB + vibostolimab	ORR: 65%, CR: 13%, DoR: 13.7 months; PFS: 15 months	NCT05007106	[66]

Anti-tumor mechanism of chemotherapies in advanced endometrial cancer

Cell Cycle Arrest and Apoptosis

Platinum-based therapies, such as paclitaxel, are known for their role in tubulin polymerization and the stabilization of microtubules. These microtubules are pivotal in mitotic processes, forming the essential structure for proper cell division. Paclitaxel exerts its therapeutic effects by effectively hindering this assembly, disrupting the mitotic spindle; this disruption results in cell cycle arrest and eventually triggers programmed cell death [67]. Moreover, recent advancements have introduced novel agents such as sulfur heteroatinoid A2 (SHetA2). When administered with paclitaxel, SHetA2 enhances the therapeutic efficacy by inducing a G1 phase cell cycle arrest, thereby halting the proliferation of cancer cells. In addition, this combination therapy promotes significant mitochondrial damage and activates caspases, crucial mediators of the apoptotic process [68]. As a result, this synergistic approach substantially enhances anti-tumor activity, opening avenues for more effective treatment of aEC.

Cytotoxicity and DNA Damage

Chemotherapeutic agents, including doxorubicin, cisplatin, and carboplatin, are powerful drugs renowned for their ability to induce significant DNA damage, ultimately resulting in apoptosis [69,70]. Doxorubicin, a potent anthracycline, intercalates between the DNA base pairs, disrupting replication and transcription processes vital for cell survival and proliferation. On the other hand, cisplatin forms crosslinks in the DNA strands, thereby obstructing DNA synthesis and function, which triggers apoptosis in malignant cells [71]. Furthermore, doxorubicin inhibits the activity of topoisomerase II, an enzyme crucial for DNA unwinding, and generates free radicals that lead to harmful DNA strand breaks, perpetuating the pro-apoptotic pathway [70]. Other chemotherapeutic agents, such as Ifosfamide, are alkylating agents that chemically modify DNA; they induce crosslinking and create strand breaks that significantly disrupt the essential processes of DNA replication and transcription, ultimately leading to the demise of cancer cells [72,73]. This intricate interplay of mechanisms highlights the multifaceted approach of chemotherapeutics in targeting and eliminating cancerous cells in aEC.

In aEC, carboplatin-paclitaxel combines DNA crosslinking (carboplatin) with mitotic arrest (paclitaxel), achieving a synergistic cell kill. Doxorubicin, once a cornerstone for metastatic EC, induces responses through DNA intercalation but is limited due to cardiotoxicity and relatively short-lived tumor growth control [74]. Overall. chemotherapy’s multi-pronged attack can induce tumor regression, but resistance often develops via enhanced DNA repair, drug efflux pumps, or cell cycle checkpoint alterations. These limitations pave the way for integrating targeted therapy to improve efficacy.

Mechanistic rationale for lenvatinib and pembrolizumab combination

LVB + PMB was strategically created to combat tumor immune evasion by blocking angiogenesis and stimulating immune responses. LVB’s inhibition of VEGF and FGF signaling significantly impacts the TME in two primary ways. By obstructing VEGF-driven neovascularization, LVB effectively “starves” the tumor and normalizes the formation of blood vessels [75]. This normalization can lower interstitial pressure and correct convoluted vessels that typically hinder lymphocyte entry, thereby facilitating the infiltration of immune cells into the tumor [76]. Additionally, LVB has been observed to counteract immunosuppression within the TME by decreasing the presence of suppressive cells such as tumor-associated macrophages (TAMs) and Tregs, partially through off-target actions such as the inhibition of colony-stimulating factor 1 receptor (CSF-1R) on macrophages [77]. It has also been found to enhance DC activity and increase the release of proinflammatory cytokines. In preclinical studies, LVB promoted the production of interferon-gamma (IFN-γ) and the polarization of M1 macrophages, shifting the environment towards an immune-reactive state [78]. PMB functions by releasing the “brakes” on cytotoxic T-cells by inhibiting the PD-1 receptor. PMB shows limited efficacy in pMMR EC, as T-cells are frequently absent from or far from these tumors due to the VEGF-enriched and immunologically “cold” environment. However, when combined with LVB, a synergistic effect emerges: LVB modifies the TME to allow better immune cell infiltration and activation, while PMB revives exhausted T-cells to target tumor cells. This combined approach leads to improved anti-tumor efficacy.

Clinically, this rationale was confirmed by the enhanced efficacy of LVB + PMB in pMMR EC, where single-agent PMB traditionally showed ORRs below 15%. In KEYNOTE-775, the ORR with LVB + PMB was approximately 31-32% in the pMMR subgroup, significantly better than chemotherapy’s ORR of around 15% [62]. Moreover, patients responding to LVB + PMB frequently exhibit profound and lasting responses, indicating that once a robust anti-tumor immune response is established, it can maintain tumor control [54]. Although no definitive biomarker has been identified to predict which patients derive the most benefit from LVB + PMB, as PD-L1 expression and TMB did not correlate with outcomes in exploratory analyses, ongoing studies are investigating whether specific angiogenic factors or immune signatures might identify likely responders [79].

**Figure 2:**
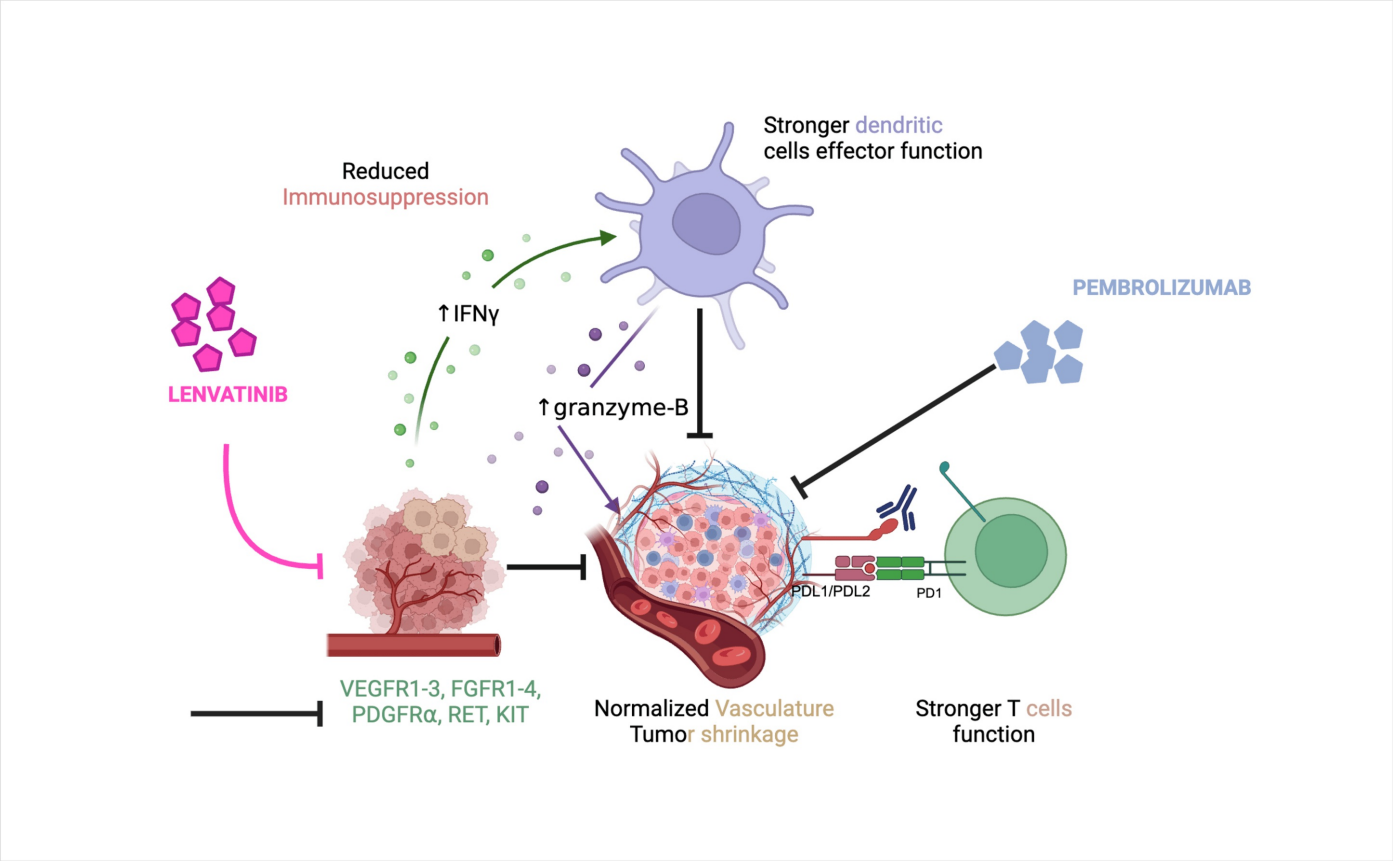
Anti-tumor mechanistic rationale for LVB + PMB Schematic diagram illustrating the complementary mechanism of LVB + PMB in the TME of advanced endometrial cancer. LVB (pink) targets multiple kinase receptors on both tumor and endothelial cells (VEGFR1-3, FGFR1-4, PDGFR⍺, RET, and KIT) to inhibit angiogenesis and growth signaling pathways. This can lead to vasculature normalization, improved perfusion, and vessel permeability. VEGF also mediates the recruitment of immunosuppressive cells (MDSCs, Tregs, M2 TAMs, tolerogenic DCs). By blocking this VEGF-driven signal, LVB reduced immunosuppression to promote antigen presentation by DCs, effector T cell function through IFN-γ, and granzymes. On the other side, PMB (blue) blocks the interaction of PD-1 on both PD-L1/PD-L2 on myeloid cells and tumor cells to release the brake on T cells and restore T cell functionality. This combined effect produces a robust anti-tumor response. This is an original figure made by the authors using BioRender. LVB, lenvatinib; PMB, pembrolizumab; TME, tumor microenvironment; VEGF1-3, vascular-endothelial growth factors 1-3; FGFR 1-4, fibroblast growth factor receptor 1-4; PDGFR⍺, platelet-derived growth factor receptor ⍺; MDSC, myeloid-derived suppressor cell; Tregs, regulatory T cells; M2 TAM, tumor-associated macrophage; DC, dendritic cell; IFN-γ, interferon-gamma; PD-1, programmed death-1; PDL1/PDL2, programmed cell death-ligand 1/2

In summary, the combination of LVB and PMB utilizes a “two-hit” approach: targeting tumor blood vessels while inhibiting immune checkpoints and effectively converting immune-cold tumors into immune-responsive ones. This method illustrates the larger rationale behind merging anti-angiogenic treatments with immunotherapy, which has demonstrated potential in EC and various other cancers, such as renal cell carcinoma. The mechanistic rationale of this combination is outlined in Figure 2. The next sections will detail the clinical effectiveness and safety outcomes of LVB and PMB compared to standard chemotherapy.

Lenvatinib/pembrolizumab versus chemotherapy: efficacy perspective

The combination of PMB and LVB has emerged as a second-line treatment for aEC. Nonetheless, its relative effectiveness compared to standard chemotherapy, particularly in first-line treatment, needs thorough assessment, considering factors such as clinical outcomes, safety, and guidelines. An updated phase III 309/KEYNOTE-775 study of LVB plus PMB in previously treated aEC showed better efficacy than chemotherapy in all select patient groups [62,63].

The effectiveness of LVB + PMB for aEC was well established and recently reaffirmed in the phase III 309/KEYNOTE-775 trial. This trial involved patients with advanced or recurrent endometrial carcinoma who had progressed after prior platinum-based chemotherapy. Significantly, around 70% of participants had pMMR disease, the primary target population for LVB + PMB, while around 30% had dMMR. The combination was compared to standard second-line chemotherapy options, including the investigator’s choice of doxorubicin or weekly paclitaxel. LVB + PMB demonstrated superior efficacy across all primary endpoints described below. OS for LVB + PMB reached a median of 17.4 months (hazard ratio 0.65, p<0.0001), surpassing that of chemotherapy, which was 12 months [62]. This marked a significant improvement in OS and established the combination as a life-extending therapy. The median PFS was 6.6 months compared to 3.8 months for chemotherapy. This benefit was consistent in both pMMR and dMMR groups. The ORR for LVB/PMB was 33.8% compared to 14.7% for chemotherapy. The DoR was also notably extended, indicating its potential for achieving more sustainable clinical outcomes. Tumor shrinkage was both deeper and durable with this combination. Furthermore, subgroup analysis revealed that patients with shorter platinum-free intervals (less than six months following first-line chemotherapy) derived greater benefits from the combination than chemotherapy [63].

Real-world data have further validated these results. For instance, Wang et al. conducted a retrospective comparison of LVB + PMB versus a repeat platinum-doublet (carboplatin-paclitaxel) in previously treated EC. They found no significant differences in median OS. However, patients with dMMR tumors experienced better survival on LVB + PMB compared to chemotherapy [80]. Another retrospective study from Taiwan indicated that LVB + PMB provided longer OS than doxorubicin in platinum-pretreated EC, particularly enhancing 12-month OS rates [74]. These findings reinforce that LVB + PMB is at least as effective as, and often superior to, traditional chemotherapy in salvage settings, especially for the biologically suitable subset of patients.

In first-line (systemic therapy-naïve) aEC, LVB + PMB was investigated as the initial therapy for aEC in a phase III ENGOT-en9/LEAP-001 trial. Unlike in the second line, their combination did not show a clear advantage over standard carboplatin-paclitaxel in an unselected population. The trial failed to meet the necessary statistical benchmarks, as the PFS and OS in the pMMR group were found to be low. For instance, the ENGOT-en9/LEAP-001 study reported a median PFS of 9.6 months for pMMR populations using LVB + PMB versus 10.2 months in the chemotherapy arm [64]. The median OS in the pMMR group was 30.9 versus 29.4 months in the LVB + PMB group. However, in the dMMR group, LVB + PMB prolonged OS, PFS, ORR, and median DoR.

Results from the first-line study suggested that LVB + PMB should not replace chemotherapy as the standard-of-care treatment in all aEC. Instead, its use in the frontline may be considered on a case-by-case basis, such as for patients unable to tolerate cytotoxic chemotherapy or possibly for certain high-risk pMMR profiles (although this is not yet established from trial data). The negative outcome of ENGOT-en9/LEAP-001 underscores the importance of patient selection and the potential advantage of combining immunotherapy with chemotherapy (rather than TKIs) in the frontline, at least for pMMR tumors.

Growing evidence suggests that molecular stratification can effectively guide the use of LVB + PMB. It has been shown that dMMR tumors respond favorably to ICIs, whether used independently or alongside chemotherapy [23,51]. For this subtype, first-line treatment with LVB + PMB is generally unnecessary, as either ICI alone or in combination with chemotherapy can achieve good outcomes with minimal side effects. Nonetheless, LVB + PMB may be appropriate for dMMR patients who have either progressed following immunotherapy or cannot undergo chemotherapy due to contraindications [81]. In contrast, for pMMR tumors, LVB + PMB has demonstrated improved outcomes, as reflected in the OS and PFS results from the KEYNOTE-775 trial. Therefore, current evidence supports its designation as the primary standard second-line treatment for pMMR aEC. In fact, international guidelines now advocate for LVB + PMB in any platinum-pretreated pMMR EC, while dMMR cases should receive ICI alone if they have not yet been treated with it.

In the context of pMMR, further categorization using ProMisE could provide valuable insights. A study conducted by Chiba et al. indicated that patients with high copy-number p53-abnormal endometrial carcinoma experienced significantly poorer outcomes when treated with LVB + PMB, exhibiting notably shorter PFS in comparison to other molecular subtypes [82]. This finding implies that tumors with p53 mutations, which often display more severe histological characteristics, might be less responsive to LVB + PMB, potentially due to their aggressive nature or through alternative and redundant resistance mechanisms. Conversely, pMMR tumors categorized as NSMP or those with particular mutations, such as PTEN, could derive some benefit from the treatment, although the available data are limited. These results underscore the importance of incorporating additional molecular classifications in future clinical trials and when considering LVB + PMB, as p53-abnormal tumors might need to be directed towards different treatment options or clinical studies.

In conclusion, LVB + PMB has demonstrated a greater efficacy than chemotherapy in second-line treatment, significantly improving PFS and overall OS for aEC patients who have already undergone first-line therapies. Its advantages are particularly pronounced in patients with pMMR disease, addressing a significant gap in this group. On the other hand, when used as a first-line treatment, LVB + PMB does not surpass standard chemotherapy, which means it is not recommended for initial treatment in all cases. The most effective use of LVB + PMB is in patients with recurrent or refractory aEC, particularly those with pMMR tumors or those who have had rapid recurrences following chemotherapy. Current studies, including those exploring the combination of LVB + PMB with chemotherapy or other immunotherapies in the initial treatment phase, could help clarify its role further. Proper patient selection, considering MMR status, molecular subtype, previous treatment responses, and overall health, is essential to fully harness the benefits of LVB + PMB.

Safety insights on lenvatinib/pembrolizumab versus chemotherapy in endometrial/ovarian cancer treatment

Chemotherapy has been used for decades in EC patients, with many of the adverse events (AEs) well recorded. The National Cancer Institute established standard definitions for AEs following cancer therapy. These definitions are known collectively as the Common Terminology Criteria for Adverse Events (CTCAE) or Common Toxicity Criteria (CTC). They help explain the grade of organ toxicity in patients receiving cancer therapy on a scale of 1 to 5, with grade 1 being mild and grade 5 usually representing the highest AEs such as death [83]. AEs from chemotherapy use in cancer are well recorded. The common AEs reported with taxane- or platinum-based chemotherapy (mostly paclitaxel, carboplatin, and cisplatin) in EC patients include nausea, neutropenia, thrombocytopenia, neuropathy, vomiting, and diarrhea [43].

The combination of LVB and PMB introduces a unique set of toxicity considerations that oncologists must address, differing from the traditional toxicities seen with chemotherapy. LVB, similar to other TKIs, is associated with a range of well-documented class-specific AEs, including hypertension, gastrointestinal disturbances, and various organ-specific toxicities [84]. On the other hand, PMB can lead to various immune-related AEs [85]. In this discussion, we evaluate the safety profile of the LVB + PMB combination, utilizing data from clinical trials and real-world studies.

Common AEs linked to traditional anthracycline-based chemotherapy include significant myelosuppression, marked by neutropenia, anemia, and thrombocytopenia [86]. This affects many patients, with grades reaching at least 3. Gastrointestinal issues, such as nausea, vomiting, and diarrhea, also frequently occur. Additional side effects can include neuropathy (related to paclitaxel), hair loss (alopecia), and fatigue. Although severe hematologic AEs (≥ grade 3) associated with chemotherapy can be serious, they are typically reversible and episodic, with treatment-related fatalities being uncommon. For example, data from GOG-209 indicate that around 50-60% of patients experienced grade 3 or higher hematologic AEs [43]. In clinical trials involving chemotherapy for EC, around 1% of patients suffered from treatment-related deaths [64]. Fortunately, many known side effects of chemotherapy can be effectively managed through supportive measures, including dose modifications, growth factors, and antiemetics.

The combination of LVB and PMB may lead to a significant toxicity profile due to their synergistic effects. In the first-line LEAP-001 trial, nearly all participants (98%) experienced at least one treatment-related AE, which is comparable to chemotherapy (97%). However, grade 3-4 AEs were notably higher in the LVB + PMB cohort [55]. Hypertension, a common AE linked to VEGF inhibition, appeared in 64% of the target population. Additionally, around 40% developed hypothyroidism, likely due to the impact of LVB on the thyroid, necessitating thyroid hormone replacement therapy. Diarrhea, potentially caused by either drug - LVB's gastrointestinal effects or PMB-associated immune-mediated colitis - was reported by approximately 40-50% of patients. Fatigue and reduced appetite were observed in around 50% of cases, while proteinuria affected around 30% of the LVB + PMB group compared to 16% in those receiving chemotherapy. Hand-foot syndrome and stomatitis were rare but noted in some individuals treated with LVB + PMB [64].

Crucially, treatment-related AEs were considerably elevated in the LVB + PMB group compared to chemotherapy. From the LEAP-001 study, 39% of patients on LVB + PMB had to discontinue treatment due to AEs in contrast to 17% in the chemotherapy group [64]. There were treatment-related deaths in 2% of patients receiving LVB + PMB compared to 1% in the chemotherapy cohort. The fatalities in the LVB + PMB group were attributed to severe vascular complications such as cerebral hemorrhage, stroke, gastrointestinal perforation, and respiratory failure - risks that, although rare, are gravely associated with anti-VEGF therapy. In the chemotherapy group, deaths were primarily due to infections such as pneumonia and sepsis.

In the KEYNOTE-775 second-line trial, which involved heavily pre-treated patients, AEs were noted in 99% of participants in both treatment groups. Grade 3 or higher AEs were observed in 89% of patients receiving the combination of LVB and PMB compared to 73% in the chemotherapy group, indicating a greater incidence of severe toxicity with the combination therapy. The most frequently reported AEs in the LVB + PMB group included hypertension (64%, with 14% having grade 3 or higher), hypothyroidism (affecting nearly one-third of patients), diarrhea, fatigue, and loss of appetite. Conversely, the chemotherapy group primarily experienced anemia (49%) and fatigue [62]. Serious AEs that necessitated hospitalization were more common in the LVB + PMB group, with febrile neutropenia being the most frequent serious AE in the chemotherapy group, while uncontrolled hypertension was a significant concern in the LVB + PMB group. In terms of mortality, 5.7% of patients treated with LVB + PMB died due to treatment-related causes, while 4.9% of those in the chemotherapy group experienced the same outcome, reflecting that LVB + PMB was associated with more severe toxicities compared to standard chemotherapy.

Data from real-world studies indicate that the toxicity associated with the combination of LVB and PMB is elevated in comparison to the chemotherapy group. Additionally, there was a higher occurrence of dose reductions when using LVB + PMB [87]. In the KEYNOTE-775 study, 66.5% of participants needed to adjust their doses, tolerating only 10-14 mg instead of the recommended 20 mg, and 33% discontinued due to toxicity [62]. However, with appropriate supportive care, it is possible to manage the toxicity effectively. It is essential to provide thorough counseling on blood pressure monitoring, managing diarrhea, handling skin reactions such as rash and hand-foot syndrome, thyroid function monitoring, and conducting regular laboratory tests to keep track of liver and kidney functions and protein levels. Proper interventions and management of side effects can prevent them from escalating to grade 3 severity.

Regarding quality of life (QoL), it is interesting to note that, despite a notable burden of toxicity, analyses from the trials suggest that QoL either remains stable or may even improve slightly for those on LVB and PMB during the initial months compared to those receiving chemotherapy. This could be attributed to the cumulative toxicities experienced by chemotherapy patients that negatively impact their everyday lives (e.g., neutropenia, thrombocytopenia) [88]. In contrast, although the side effects of LVB and PMB can be long-term, they can be effectively managed and resolved over time. In fact, patients who continued with LVB and PMB had a median DoR of 14.4 months in comparison to 5.7 months for those on chemotherapy in the KEYNOTE-775 trials, demonstrating the efficacy of the treatment and patients’ willingness to adhere to the therapy with the necessary dose modifications.

Retrospective studies in real-world settings offer valuable insights as well. According to Tochigi et al., 13 out of 15 patients experienced a need for dosage adjustments due to AEs, and every patient reported some form of side effect. Hypertension and hypothyroidism were the most frequently observed reactions, affecting almost all patients, while one individual developed grade 4 immune-related pneumonitis that necessitated intensive care hospitalization [89]. There have also been reports of some uncommon but significant side effects, such as grade 2 esophagitis associated with LVB + PMB, which improved with a lower dose [90]. Additionally, radiation-recall dermatitis was noted, leading to severe skin inflammation in previously irradiated areas, but this completely resolved after discontinuation of LVB [91]. Instances of hepatic toxicity and renal dysfunction have been noted intermittently as well.

In summary, when compared to chemotherapy, the safety profile of LVB combined with PMB is notably different, primarily marked by metabolic and vascular AEs (such as hypertension and thyroid dysfunction), gastrointestinal issues, and certain immune-related effects. In contrast, chemotherapy is associated with hematologic and neuropathic AEs. Both treatment regimens necessitate careful monitoring and management, but oncologists should be particularly vigilant regarding blood pressure, treatment of diarrhea, and dosage adjustments in response to signs of toxicity. While LVB + PMB may require a greater level of monitoring for toxicity compared to chemotherapy, it provides significant clinical benefits that can warrant the additional effort in suitable patients. Establishing standardized guidelines for managing toxicity, as proposed by Zribi et al. [88], will be crucial for assisting clinicians in handling side effects. Ongoing trials such as OPTI-DOSE (NCT05949424) are assessing optimized dosing schedules (e.g., reduced or intermittent dosing) to enhance tolerability. These approaches aim to sustain efficacy while minimizing AEs.

Discussion and clinical implications

The comparison between LVB + PMB and chemotherapy in aEC has significant clinical ramifications. Notably, the demonstrated effectiveness of PMB + LVB in second-line therapy has redefined the standard treatment for patients with pMMR tumors [62]. In cases where chemotherapy yields only a limited response, the combination of LVB + PMB leads to a marked increase in OS and improved RRs. Consequently, various international guidelines, including the European Society for Medical Oncology (ESMO) and NCCN, now recommend LVB + PMB as a preferred treatment for platinum-pretreated dMMR aEC patients [92]. This allows for easy categorization of patients: those with dMMR are directed toward immunotherapy or chemotherapy combined with ICIs (chemo-ICI) at the initial treatment stage. In contrast, pMMR patients receive chemotherapy first, followed by LVB + PMB [93]. Therefore, it is crucial for healthcare providers to ascertain the MMR status of a patient’s tumor prior to initiating treatment. This tailored approach not only enhances the effectiveness of the intervention but also minimizes the risk of unnecessary side effects.

The findings from the LEAP-001 trial highlight that LVB + PMB does not show a clear advantage over chemotherapy as a first-line treatment, underscoring the ongoing importance of chemotherapy in upfront management, particularly for patients with pMMR [64]. While the disappointing outcome was unexpected, especially given the strong performance of LVB + PMB post-chemotherapy, it may stem from the observed efficacy of chemotherapy in various EC patients [43], which is reflected in a favorable PFS in the control group. This is particularly true for pMMR patients, who may respond positively to chemotherapy despite being resistant to immunotherapy. Furthermore, the increased toxicity associated with LVB + PMB in the initial treatment stages could potentially hinder its effectiveness. Moreover, the patient selection for the LEAP-001 trial was inclusive of all patients regardless of their molecular subtype, suggesting that certain high-risk groups might find LVB + PMB beneficial [64]. Therefore, until more conclusive data are available, immunotherapy (ICI) by itself or in combination with chemotherapy for dMMR tumors, or chemotherapy alone for pMMR patients, should continue to be the standard first-line treatment, while LVB + PMB should be considered for later stages of therapy.

Recent findings indicate that personalized treatment strategies are becoming essential for addressing aEC. The traditional approach of applying a standard chemotherapy regimen is becoming obsolete. It is crucial to consider risk stratification based on MMR status as we advance towards integrating comprehensive molecular classification methods, such as ProMisE and TCGA, into clinical decision-making. For example, a patient diagnosed with POLE-ultramutated metastatic EC may have better outcomes with immunotherapy as a standalone treatment [94,95]. Conversely, a patient with aggressive p53-abnormal aEC may require more intensive treatment strategies, which could include the addition of trastuzumab for HER2-positive cases or exploring clinical trials utilizing triplet therapies (combinations of chemotherapy with dual immunotherapy or chemotherapy with ICIs and TKIs) [26,96]. The observation that p53-mutant tumors exhibit poor responses to LVB and PMB highlights the need for alternative therapeutic options, such as incorporating PARP inhibitors in patients with homologous recombination deficiencies or adding CTLA-4 inhibitors to enhance immune response.

The fourth point to consider is the rapid growth of biomarker-driven therapies. Already, MMR and HER2 have been established as actionable biomarkers for EC. Moving forward, potential biomarkers might include the density of TILs or specific immune gene signatures [97]. A tumor with high immune infiltration and pMMR, which might suggest it is immune-excluded due to factors such as VEGF, could particularly gain from the addition of an anti-VEGF treatment such as LVB. On the other hand, a tumor characterized as an immune desert may require different strategies that go beyond anti-PD-1 or anti-VEGF therapies. Angiogenic markers, including Ang2 and VEGF levels, could help pinpoint tumors that rely heavily on angiogenesis, making them potential candidates for LVB [98]. The phase II study on LVB pointed to Ang2 as a possible predictive marker, but it has yet to be integrated into standard clinical practice [98].

Furthermore, specific mutations such as PTEN and catenin β-1 (CTNNB1) might play a role in determining the success of immunotherapy. For example, evidence has suggested a connection between CTNNB1 and TME that is immune-cold in solid tumors, which could signify a poor response to ICIs [99]. If this link is validated, these patients may be stratified for upfront treatment with LVB + PMB, even if they are dMMR or involved in novel clinical trials. From a clinical practice perspective, oncologists must be prepared to manage aEC patients over a longer term, often on continuous daily therapy rather than intermittent chemotherapy cycles. This means managing chronic toxicity, monitoring blood pressure and thyroid function, etc., become as important as tumor imaging. Close follow-up is advised when starting LVB + PMB, and early dose adjustment could help mitigate AEs.

In conclusion, interdisciplinary collaboration is crucial; for instance, cardiologists manage hypertension, endocrinologists handle thyroid problems, and palliative care specialists focus on symptom relief. Although there are obstacles, the benefits are significant: patients, even those in hospice after chemotherapy, are experiencing longer lifespans and better outcomes due to effective disease control with LVB + PMB. The treatment of advanced aEC has progressed with LVB + PMB, particularly for patients with pMMR. The challenge ahead lies in identifying the right candidates for this combination therapy, determining the optimal timing for administration, and effectively managing potential side effects while integrating it with treatments such as chemotherapy or immunotherapy. According to current research, our recommended approach is to start with carboplatin/taxane chemotherapy, adding PMB or dostarlimab for those with dMMR; for the second-line, for pMMR patients (or dMMR patients who have already undergone immunotherapy), consider LVB + PMB due to its proven survival benefits; meanwhile, dMMR patients who have not had ICIs should be given single-agent PMB or dostarlimab. Beyond the second line, pursuing clinical trials or personalized therapy may be appropriate, which could include administering LVB + PMB if it has not been previously utilized, or exploring other targeted treatments depending on specific markers. Each treatment decision should be tailored to the patient, considering their previous treatments, molecular characteristics, existing health conditions, and preferences, particularly after discussing the various toxicity profiles. This tailored approach will enable patients to gain the greatest advantage from the widening range of therapies available for aEC.

Potential biomarker of personalized medicine in endometrial cancer

Personalized medicine is increasingly vital in EC due to its molecular heterogeneity. Identifying biomarkers that can predict a patient’s prognosis or response to treatment is essential for tailoring therapies to individual needs. Over time, numerous significant and emerging biomarkers have been identified for aEC. Key biomarkers among these are discussed below.

The MMR status (dMMR or pMMR) is currently the most significant biomarker in clinical practice. MMR tumors, which make up around one-third of aEC cases, show a high sensitivity to PD-1 inhibitors, while pMMR tumors do not exhibit the same response [32,100]. Consequently, routine MMR status testing is now standard for all EC patients [32]. This information is crucial for determining the appropriate first-line treatments (chemotherapy and/or ICIs for dMMR patients, chemotherapy alone for pMMR) and second-line options (ICI monotherapy for dMMR if it has not been administered already, and LVB + PMB for pMMR). MMR status also serves as a prognostic indicator: dMMR tumors tend to respond slightly better to immunotherapy compared to pMMR tumors. Historically, dMMR tumors have shown a somewhat worse OS rate, but with the introduction of immunotherapy, their prognosis has significantly improved [23,101].

 An ultramutated *POLE* gene is a biomarker indicating high immunogenicity and a favorable prognosis. Tumors with POLE mutations often achieve complete responses to standard therapies and are likely to benefit from ICIs as well [102]. Although POLE mutations are relatively infrequent (~5-10% of EC cases), recognizing them can impact treatment strategies. For example, some studies have investigated reducing adjuvant treatment for early stage POLE-mutant EC due to their promising outcomes [103]. In advanced EC, a POLE-mutant pMMR tumor may still respond well to immunotherapy, likely because of a high neoantigen load closely resembling the MSI-H phenotype [104]. Therefore, assessing POLE status through molecular or sequencing tests is valuable in clinical practice.

TMB is variable in EC. Tumors classified as MSI-H or POLE ultramutated have extremely high TMB and usually show favorable responses to ICIs [105]. Interestingly, even some pMMR ECs can exhibit moderately high TMB due to specific driver mutations such as ARIDIA, which are linked to genomic instability [106]. The FDA has authorized PMB for any tumor exhibiting TMB > 10 mutations per megabase if no alternative treatment options are available [107]. In the context of EC, TMB is not commonly assessed outside of research settings since evaluating MSI status is a more straightforward alternative. Nevertheless, redefining the classification to include terms such as “hypermutated but MSI-stable” could uncover additional patients who may benefit from immunotherapy or combined treatment approaches.

The p53 status, indicative of high copy number, is identified through immunohistochemistry and serves as a marker for this particular molecular subtype. Clinically, p53-abnormal EC tend to be aggressive and frequently display resistance to chemotherapy, which has also been observed in the LVB + PMB trial [108]. Understanding p53 status can aid in identifying patients who may benefit from HER2 testing, as aggressive carcinomas often demonstrate p53 abnormalities, with around 30% showing HER2 amplification. For those who are HER2-positive, incorporating trastuzumab into chemotherapy regimens can enhance outcomes [108,109]. Furthermore, patients with p53 mutations may be directed towards clinical trials that involve intensified treatment strategies, such as adding CTLA-4 or PARP inhibitors, since these tumors often require a more prolonged treatment approach. In clinical practice, pathologists typically report p53 IHC findings in EC cases, enabling oncologists to make informed decisions regarding trastuzumab treatment (in cases of serious or HER2-positive cancers) or designating patients as high-risk, warranting more diligent follow-up care [57,109].

The status of estrogen receptors (ERs) and progesterone receptors (PRs) serves as a traditional biomarker in EC. Tumors that are positive for ER/PR, which are typically of the endometrioid type and often fall into the NSMP category, can be treated with hormonal therapies, including progestins and aromatase inhibitors [110]. This is particularly relevant for low-grade atypical aEC or for patients looking to maintain their fertility. In more advanced situations, while hormonal treatments may have limited effectiveness, they can be paired with targeted therapies. For instance, the combination of everolimus (mTOR inhibitor) and letrozole (aromatase inhibitor) has shown some promise in clinical activity [111]. Moreover, positivity for hormone receptors generally indicates a more favorable prognosis [110]. As a result, understanding a patient’s ER/PR status can influence treatment decisions for hormonal therapy - either as a low-toxicity maintenance option (some oncologists continue prescribing megestrol acetate or letrozole for responding ER-positive patients’ post-chemotherapy) or for providing palliative care in less fit patients. Tailored treatment would involve utilizing hormone therapy for those likely to respond (such as patients with strong ER/PR positivity and low-grade histology) while steering clear of potential side effects in individuals unlikely to gain benefits (like those with ER/PR negativity and high-grade tumors).

Various gene mutations are frequently associated with EC, but only a handful are currently actionable. PTEN mutations occur in around 50% of endometrioid EC cases, while ARIDIA mutations occur in approximately 30%, and both have been linked to improved outcomes when treated with avelumab immunotherapy [27]. This might be due to their ability to increase the immunogenic nature of the TME through the activation of the PI3K pathway or the formation of neoantigens [112]. CTNNB1 mutations (particularly prevalent in NSMP tumors) occur in around 20% of EC cases and are under investigation as a potential negative predictor for response to ICIs, attributed to an “immune-excluded” TME [113]. Additionally, FGFR2 mutations, found in around 10% of EC cases, suggest the possibility of targeted treatment options such as erdafitinib; preliminary reports show sporadic positive responses to FGFR inhibitors [114]. Current basket trials are examining FGFR inhibitors across various FGFR-altered solid tumors, including EC. In a personalized medicine context, genomic sequencing of recurrent or atypical endometrial carcinoma can uncover treatable mutations, allowing patients to access off-label targeted therapies or appropriate trial options.

Besides the intrinsic traits of tumors, elements within the TME can act as biomarkers. For instance, increased levels of VEGF-A or serum ANG2 may indicate the existence of tumors driven by angiogenesis, which are likely to show significant responses to anti-VEGF treatments across different cancer types [115,116]. Additionally, a profile that shows an immune microenvironment marked by high PD-L1 levels, CD8+ TILs, and an IFN-γ gene signature is often linked to better responses to immunotherapy in cancer patients [117]. In the case of EC, the effectiveness of PD-L1 IHC as a predictive marker is somewhat restricted, partly due to the heterogeneity of tumors [24]. Nonetheless, a composite immune score could provide important insights. One research study identified an “immune-high” subgroup within endometrioid tumors (not necessarily MSI-H) with a substantial presence of TILs and markers indicating checkpoint expression, highlighting their potential as candidates for ICIs [118]. With the progress in bioinformatics, analyzing tumor RNA sequencing might enable the establishment of a score that forecasts the potential advantage of immunotherapy compared to anti-angiogenic therapies. This could guide decisions on whether to use PMB as a standalone treatment, in combination with LVB, or other therapeutic alternatives. The ultimate goal of personalized medicine is to integrate these biomarkers to customize treatment plans for individual patients. Although this has not been fully realized yet, many biomarkers are still undergoing investigation. However, the ProMisE classifier is actively used in tumor boards for case discussions [119]. As more outcome data becomes available, it is expected that treatment approaches will begin to incorporate these molecular profiles, paving the way for clinical trials that specifically investigate these important issues soon.

Research direction and future perspectives

The introduction of LVB combined with PMB marks a significant advancement for aEC, but several questions and potential avenues for research persist. Future studies should concentrate on key areas, including the best combination and order of current and newly emerging treatment options. One hypothesis is that initiating with a triple therapy approach, specifically chemotherapy in conjunction with ICI and LVB, could enhance outcomes for pMMR patients. The DUO-E trial already evaluated this possibility with chemotherapy (paclitaxel/carboplatin) plus durvalumab with or without olaparib and showed improved PFS [56]. Nevertheless, while this method may show promise, it is crucial to weigh its effectiveness against the risk of increased toxicity; should this triplet therapy be too harmful, an alternative strategy could involve a sequential approach. In this case, initial chemotherapy could be used to reduce tumor burden and slow rapid progression, followed by a transition to LVB and PMB for ongoing maintenance treatment. This could allow for a beneficial management of overlapping toxicities. Further trials or retrospective studies might help examine this approach [120].

Expanding the scope of immunotherapy beyond just PD-1 blockade is crucial. Other checkpoints such as CTLA-4 inhibitors (e.g., ipilimumab) and LAG-3 inhibitors (e.g., relatlimab) may enhance immune responses when used alongside PD-1 inhibitors. This is partly due to the distinct roles these agents play in the activation and functionality of T cells, where ipilimumab acts during the activation stage and PMB functions during the effector stage. A notable example is the KEYVIBE-002/005 trial, which combines favelizumab (an anti-LAG3 agent) with PMB and/or LVB, and also evaluates the combination of vibostolimab (an anti-TIGIT agent) with PMB and LVB in dMMR EC, showing promise for effective outcomes [65,66]. Additionally, ongoing investigations into bispecific antibodies targeting both PD-1 and CTLA-4, such as cadonilimab, are expected to reveal whether pairing with LVB can benefit non-responders or enhance the response of partial responders into full responders [58].

The importance of biomarker-driven trials cannot be understated, as they play a critical role in patient stratification and enrollment restrictions based on molecular characteristics. This is supported by evidence from the DUO-E trial, which demonstrated that incorporating a PARP inhibitor with durvalumab maintenance therapy improved PFS. Future research could include more biomarker-based stratification approaches, such as examining BRCA or PALB2 mutations (though these are relatively uncommon in EC), to better understand the effects of PARP inhibitors [30]. Additionally, for patients whose tumors demonstrate positivity for the PI3K mutation pathway, PI3K-ARK-mTOR inhibitors might be evaluated in combination with ICIs LVB [121]. Recent advancements in proteomics, especially high-resolution and comprehensive platforms, provide unprecedented insights into the molecular complexity of diseases [122]. This technology enables the identification and quantification of tumor-specific protein signatures, post-translational modifications, and dysregulated pathways that might not be apparent through genomic profiling alone. By integrating genomic, proteomic, and transcriptomic information, clinicians and researchers can more effectively stratify patients, predict therapeutic responses, and discover novel biomarkers for treatment and monitoring recurrence.

Addressing resistance has become increasingly important as more patients are treated with ICIs, either alone or in combination with LVB. This may lead to the emergence of new resistance patterns, particularly if treatments are initiated early in the disease process. Consequently, further investigation is necessary to understand the mechanisms behind resistance to LVB in conjunction with PMB. Potential mechanisms may include the increased activity of alternative angiogenic pathways or the activation of immunosuppressive pathways, including the upregulation of CTLA-4 and TIM3 on T cells. Additionally, changes in tumor genetics, such as the loss of beta-2 microglobulin, which leads to decreased expression of major histocompatibility complex (MHC) class I and subsequent immune evasion, should also be considered [123-125]. Moreover, understanding the mechanisms behind resistance and subsequent treatment options is still lacking. When tumors progress while on LVB + PMB, determining the next step remains a challenge. At that point, the tumors would likely have been previously exposed to both chemotherapy and ICI. Alternative strategies might involve targeting other mutations or changes in the TME that contribute to resistance. For instance, if resistance is linked to the upregulation of alternative angiogenic pathways, switching to different TKIs or incorporating MET inhibitors in cases of MET activation could be viable options [126]. However, such hypotheses necessitate biopsy-driven studies at the time of disease progression.

At the same time, efforts in innovation should prioritize maintaining treatment effectiveness while reducing adverse effects. The OPTI-DOSE trial aims to discover the minimum effective dose of LVB for patients who typically find it challenging to manage the standard dose of 20 mg. Starting with a lower dose, such as 14 mg, or implementing an intermittent dosing strategy may support preserving PFS benefits while decreasing adverse side effects [127]. The goal is to ensure that patients do not stop a helpful treatment because of undesired adverse reactions. Additionally, future studies on factors predicting toxicity, such as initial characteristics indicating the risk of developing hypertension or liver-related complications while on LVB, could enable timely modifications in treatment approaches.

In conclusion, addressing the existing research gaps is essential, particularly the absence of validated predictive markers for determining which patients might benefit from LVB. Unlike certain cancers where specific mutations or PD-L1 expression guide the use of TKI therapies, LVB treatment for EC generally treats all patients with pMMR tumors similarly. Identifying reliable biomarkers, such as serum ANG2, could be significantly beneficial in pinpointing patients likely to derive greater advantages from LVB combined with LVB + PMB [128]. For instance, the retrospective analysis from the KEYNOTE-146 correlative study failed to identify such predictive biomarkers, as it showed that all patients experienced some level of benefit, regardless of TMB or genetic mutation profiles [79]. Exploring larger datasets or conducting combined analyses might provide further insight into this issue.

Furthermore, the optimal dosing regimen for LVB + PMB remains uncertain. In clinical trials, patients were treated until they experienced either disease progression or unacceptable side effects. However, for those who achieve a complete response, the question arises: is it necessary to continue treatment indefinitely, or should therapy be halted after a certain remission period, akin to some protocols in melanoma immunotherapy? This aspect has yet to be clearly defined, and prolonged treatment could lead to increased costs while potentially exacerbating chronic toxicities. Future trials designed to discontinue treatment after a specified period and resume it upon disease progression could provide relevant insights.

**Figure 3:**
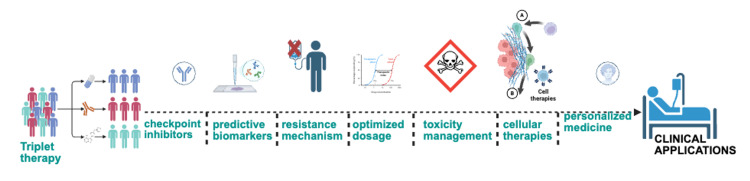
Future direction for translational approach in aEC The figure highlights several strategies to enhance the treatment regimen for aEC and to develop new effective therapies. These strategies include the design of triplet therapy targeting multiple pathways, the use of additional checkpoint inhibitors that are highly expressed by the tumor, methods to address resistance mechanisms or compensatory pathways, optimizing dosages to minimize toxicity while maintaining efficacy, incorporating cellular therapies (such as CAR-T, TILs, BiTEs, and TCR-T cells), and adopting personalized medicine approaches based on tumor characteristics, patient history, overall fitness, immune composition, etc. All these strategies aim to improve the clinical effectiveness of treatments in aEC. aEC, advanced endometrial cancer; CAR-T, chimeric antigen receptor T-cell; TIL, tumor-infiltrating lymphocytes; BiTEs, bispecific T cell engagers; TCR-T, T cell receptor T-cell

As we look into the future, we expect that the results will lead to further incorporation into existing guidelines and practices, particularly about GY018 and DUO-E’s OS final analysis [10,56,129]. Essentially, the future points towards a multi-modal, biomarker-focused strategy for aEC. Clinicians will be skilled at understanding molecular testing and selecting from various treatment options. Cooperation among gynecologic oncologists, medical oncologists, molecular pathologists, and researchers will be essential to optimize treatment combinations. An exciting prospect on the horizon is personalized immunotherapy, which may involve creating vaccines or T cell treatments targeting neoantigens in EC (especially those with ultra-mutated tumors). Early clinical trials are investigating adoptive T cell therapy aimed at cancer-testis or mutated antigens in solid tumors such as EC. If these approaches prove successful, they could offer new hope for patients who have not responded to current therapies. An all-inclusive trial design is crucial to prioritize intentional methodologies that accurately reflect the true representation of EC tumor biology. This is especially important for populations that are often misrepresented or underrepresented, such as those in Africa [130]. Hence, moving forward, the emphasis will be on intelligent integration, reducing toxicity, and matching the most appropriate treatment to each patient. The summarized future direction approach is highlighted in Figure 3.

## Conclusions

aEC has evolved from primarily being treated with chemotherapy to now being addressed through immunotherapy and targeted treatments, and the combination of LVB and PMB has proven effective as a second-line therapy, bringing renewed optimism for patients. This pairing highlights the importance of understanding tumor biology, specifically the synergy between anti-angiogenic agents and ICIs, and its potential to achieve significant clinical outcomes. Likewise, first-line treatments combining chemotherapy and immunotherapy (NRG0GY018, RUBY) have transformed the care landscape for patients with dMMR. Recent findings underscore critical aspects, such as stringent patient selection criteria, the establishment of LVB + PMB as the new standard for platinum-treated aEC, especially in pMMR cases, the significance of managing toxicity for success, the emergence of combinatorial approaches, and new biomarkers, that will lead to further advancements in personalized and effective treatments.

As we look into the future, ongoing and upcoming clinical studies will enhance our treatment strategies. We will likely see a rise in triplet therapies for specific patient subgroups, innovative combinations of immunotherapy for challenging tumors, and the development of biomarker tests to determine the most suitable treatment for each individual. Clinicians should adopt a multidisciplinary and personalized approach, ensuring thorough molecular testing and educating patients on the possible side effects of newer treatments. It is crucial to collaborate closely with supportive care teams to improve the tolerability of treatments. In conclusion, although aEC remains a serious and life-threatening illness, recent advancements in therapy have significantly improved patient outcomes. Ongoing research and innovation are essential to address the remaining challenges in treating aEC. The achievements highlight the impact of integrating clinical understanding with scientific progress - an approach expected to gain momentum in the coming years.
